# Numerical Assessment of Zebra-Stripes-Based Strategies in Buildings Energy Performance: A Case Study under Tropical Climate

**DOI:** 10.3390/biomimetics7010014

**Published:** 2022-01-12

**Authors:** Miguel Chen Austin, Kevin Araque, Paola Palacios, Katherine Rodríguez Maure, Dafni Mora

**Affiliations:** 1 Research Group Energy and Comfort in Bioclimatic Buildings, Faculty of Mechanical Engineering, Universidad Tecnológica de Panamá, Av. Domingo Díaz, Panama City 0819, Panama; miguel.chen@utp.ac.pa (M.C.A.); kevin.araque@utp.ac.pa (K.A.); paola.palacios@utp.ac.pa (P.P.); katherine.rodriguez8@utp.ac.pa (K.R.M.); 2 Centro de Estudios Multidisciplinarios en Ciencias, Ingeniería y Tecnología (CEMCIT-AIP), Av. Domingo Díaz, Panama City 0819, Panama; 3 Sistema Nacional de Investigación (SNI), Clayton City of Knowledge Edf. 205, Panama City 0819, Panama

**Keywords:** biomimetics, building performance simulations, indoor thermal comfort, reflective nature, zebra stripes

## Abstract

Urban growth has increased the risk of over-heating both in the microclimate and inside buildings, affecting thermal comfort and energy efficiency. That is why this research aims to evaluate the energy performance of buildings in terms of thermal comfort (operative temperature (OP) levels, satisfied hours of natural ventilation SHNV, thermal lag), and energy efficiency (roof heat gains and surface temperatures) in an urban area in Panama City, using superficial-heat-dissipation biomimetic strategies. Two case studies, a base case and a proposed case, were evaluated using the Designbuilder software through dynamic simulation. The proposed case is based on a combined biomimetic strategy; the reflective characteristics of the Saharan ant applied as a coating on the roofs through a segmented pattern such as the Zebra’s stripes (one section with coating, and another without). Results showed that the OP decreased from 8 to 10 °C for the entire urban zone throughout the year. A reduction of 3.13% corresponding to 8790 kWh per year was achieved for cooling energy consumption. A difference of 5 °C in external surface temperature was obtained, having a lower temperature in which the biomimetic strategy was applied. Besides, it was evidenced that a contrasted-reflectivity-stripes pitched roof performed better than a fully reflective roof. Thus, the functionality of Zebra stripes, together with the reflective characteristics of the Saharan ant, provide better performance for buildings’ thermal regulation and energy needs for cooling.

## 1. Introduction

In the last century, there has been a high growth of urban areas where deforestation of green areas was not considered, and materials were used in buildings with high thermal retention, soil occupation, and high levels of greenhouse gas emissions. The greenhouse effect is on the other hand; urban areas consume between 60% and 80% of the energy produced, which today has generated a high environmental and energy cost for the planet [1].

Such consequences are reflected in the effect of urban heat islands (UHI) in cities that cause high rates of heat and temperature in urban areas and consequently affect people’s comfort both outside and inside buildings and in the high rates of energy consumption of a part of the buildings. Latin America and the Caribbean, according to United Nations studies in 2018, had one of the highest percentages of urban population with around 81%, a trend which will not decrease [2]. Panama is no exception—there is a high uncertainty regarding energy sustainability due to the growing urbanization that has left aside parameters such as interior comfort, leading to excessive use of air conditioning systems and high levels of energy consumption [3].

Recently in the country, measures have been taken regarding regulations to be able to seek options and strategies that allow greater comfort and low energy consumption in new constructions, such as the sustainable construction manual (RES), which represents the first steps in the search for new sustainable buildings with low energy consumption [4]. Estimates by the National Secretary of Energy (SNE) project that by the year 2050, 66% of homes will have at least one air conditioning unit, which means that for that year, 55% of residential consumption will be due to the use of this equipment [5]. This is mainly due to the constant increase in temperatures, as revealed in a study focused on urban heat islands in Panama City [6]), where it was identified that the materials used in constructions and roads have a high accumulation of heat, which generates a higher temperature and an increase in the use of these devices. There is a need for ventilation and to search for other types of strategies such as bioclimatic or biomimetic [7]. In addition, a study has been carried out focused on how natural ventilation can influence a favorable climate for the indoor thermal comfort and the energy efficiency of buildings in a microclimate of Panama City via dynamic simulations coupling ENVI-met and Designbuilder software [8]. Here, it was concluded that passive use would only represent 19.41% of comfort throughout the year, which is why the use of materials in buildings that allow greater thermal insulation to improve these conditions was recommended. Moreover, the use of standard weather data based on typical meteorological conditions to perform the dynamic simulations in Designbuilder was contrasted using microclimate data instead generated via ENVI-met. An average difference of about 5.07% (with a maximum of 9.79% (940 kWh) in March and a minimum of 0.9% (121 kWh) in January) was encountered when using microclimate data instead of standard weather data for the case study, leading to the consideration of microclimate data for the study of natural ventilation proficiency and the energy efficiency of individual buildings at an urban scale.

On the other hand, the search for strategies to be more efficient in buildings has led to multiple bio-inspired strategies or concepts such as biomimetics, which refers to the search for strategies in nature and how to extract these concepts to solve a human problem [9], in applications for buildings with the purpose either of developing or improving air conditioning systems [10].

In bioinspired design specifically for buildings, it is common to adopt the first level of biomimetics that studies the organism—specifically the plants as the protagonists—and the study of their adaptive behavior—precisely their shape [11].

For energy management in buildings, skins have been implemented in buildings due to biomimetic design abstraction. All the natural aspects that influence nature in the body are considered, and the natural skin is assimilated with the skin of the building, for example, membranes and organs as if they were the mechanical or electrical system of the building. Likewise, the reactions between light, humidity, air, sound, heat, and other factors influence real life. Under this aspect, there is a case study of the construction of the skin to reduce energy consumption [12] based on a biomimetic design matrix that adopts characteristics of different organisms in nature.

The first case is The Council House 2 [12], in Australia that emulated the bark of a tree; the north and south facades represent the bronchi of the tree, the west is the epidermis, the east and the facade that represented the nucleus was the bark which acted as a filter for light and moisture. The second case is the Water Cube aquatic center in Beijing [12], which through its skin emulates soap bubbles that can reduce surface area and energy, in which a specific geometric shape is obtained that is replicated in the edification. The results of this application include energy cost reduction by 30%, reduction of artificial lighting by 55%, and solar energy is trapped, serving as heating.

In the search for optimal conditions for the occupants, the importance of the study of thermoregulation [13] is highlighted, in which its authors emphasize the types of thermoregulation mechanisms for hot and cold climates and the models found in nature. Such as in termite mounds, the heat exchange of the prickly pear, and even humans themselves when sweating. Under this concept, an evaporative cooling system for building envelopes (Stoma Brick) was designed, based on four parts that make it up: in which the stoma brick mainly controls the entry and exit of moisture and at the same time retains it for evaporation. The design is adaptable for hot, cold, humid, and dry climates, mainly simulating a plant’s stomata, conifers, and human skin.

Moreover, other strategies based on skin characteristics can be found in nature for hot climates, such as the reflective properties of the Saharan silver ant’s hair [14], the head position of the Western Reef Heron [15], and the Shell in Snails and slugs [16]. Regarding the reflective strategy for heat dissipation, such as the Saharan ant [17] and the Shell in Snails [18], there are studies that focus on the application of reflective coatings on ceilings and pavements to cool down the surrounding air temperature in urban areas for urban heat island effect mitigation [19,20], a strategy called “cool roofs.” A study carried out in [21], based on simulations for cities in California with a Mediterranean climate, where the albedo of all surfaces, both pavement and ceilings, was increased by 0.2, obtaining benefits of up to −0.01$ per year per square meter of energy savings, and CO_2_ reductions of less than 1 kg per square meter. However, this type of strategy depends a lot on the climatic zone and also has its disadvantages with respect to the application on floors or ceilings, since it can affect the visibility of people and even increase the temperature of the surfaces of some buildings of greater height to others of less height [22]. Another study applied a reflective coating using light tones combined with phase change materials (PCM) to reduce the monthly cooling energy consumption. Being in a tropical area—Singapore—a reduction of cooling energy between 5% to 12% was achieved throughout the year [23]; supporting results were later obtained in [24].

The thermoregulatory functions associated with the stripes in Zebras’ skin also fall within such type of strategy. However, by mimicking the animal body via colored metal barrels, experimental evidence showed no significant difference in the core temperature of barrels covered with horse and Zebra skins with different scratch patterns. Thermographic measurements were employed. It was concluded that the stripes’ coloring and patterns do not statistically significantly influence the body thermoregulation in Zebras [25]. In contrast, a study carried out in Kenya also performs thermographic experiments on live Zebras for seven hours a day. It was evidenced that there is a temperature difference of 12 to 15 degrees between contrasted stripes [26], mainly due to sweat that is accelerated by latter, which in turn, with the movement of the air, causes turbulency at the Zebra’s hair, increasing cooling by evaporation.

On the other hand, studies have been carried out that involves the biomimetic design in facades specifically applied to insulators, which are simulated in TRNSYS to evaluate the energy reduction potential compared to having no strategy and thus obtaining a significant reduction in the greenhouse gas emissions in the useful life of the building, in which it applies for different types of climates, in different types of buildings [27]. It was recognized that the thermal bioarchitectural framework was valid as a bridge between architecture and biology [28] in order to find designs with efficient thermal performances. In the case of two buildings in New Zealand, in which the buildings were first simulated to recognize the main problems and divide the thermal zones, different scenarios were analyzed in which mainly passive and active techniques were used in combination. The authors concluded that imitating biological forms so far does not seem to be critical to assessing energy efficiency in buildings [29].

It is common to evaluate the potential ideas that biomimetic-based design can offer us. With a solution-based approach [30] these are generated under three main categories (biological domain, transfer, and technological domain) [31], multiple proposals for designs of more efficient buildings. In the review, as a first point, an extensive investigation of all the characteristics and biological behavior is carried out, taking aspects such as: heat control, use of organic material, respiratory control, use of water, among others. This knowledge is transferred and compared with similar processes in construction and engineering. For example, breathing control compared to ductwork, water usage compared to evaporative cooling. Finally, there is the technological domain in which they evaluate which tool or method can be implemented or improved in the design, be it at the level of the building, systems, or components. It was proposed to change load and energy consumption through passive design to retain and release heat, heat sinks emulating the toco-toucan, and develop new heat recovery systems based on aquatic species and products that reduce the range of current detectors by copying the behavior of some insects. The researchers highlighted the importance of bioinspired design and its potential in the future [32].

Finally, while there are multiple proposals focused on the interior conditions of individual buildings, few studies can be found that investigate the biomimetic approach at urban scale via experimental approaches [33,34,35] and even fewer via dynamic simulation [36] at this scale under tropical climates. Based on our particular interest in dynamic simulation and numerical studies, by focusing on the heat island problem mitigation, an extensive investigation of the biological analogies was carried out for a case study of the problem for the Old Town, Casco Antiguo, in Panama City [36]. Here, to evaluate and improve the thermal comfort of pedestrian (outdoor comfort) through the ENVI-met software, considerable changes were made to the roof of the buildings, emulating the physiology of Zebras and the characteristics of the Saharan ant, in addition to changes in the pavement and vegetative growth. The changes made resulted in temperature reductions of up to 4 °C in a specific area of the study and reductions in the Physiological Equivalent Temperature (PET) comfort index indicators for pedestrians. However, this study did not evaluate the impact of such biomimetic strategies in the indoor thermal comfort, envelope performance, and energy consumption.

Therefore, the objective of the present study is based on the research of reference [8], [36]. Such previous studies, performed for the same studied urban zone, concluded the following:

1. The urban heat island effect, evidenced by the external comfort, is increased by the narrowness of buildings within the urban zone under such a tropical climate [36];
2. Natural ventilation (or passive mode) appears not viable, and the extensive use of air conditioning systems (or active mode) may be required to provide acceptable indoor thermal comfort [8];
3. Other thermoregulation strategies are needed to improve external comfort [36];
4. The use of microclimatic data could significantly influence the estimation of the building energy performance [8], causing about a 10% difference lower for cooling needs than when using standard weather data.

Since [8] evaluated the passive and active modes in the base case study (with no incorporation of biomimicry) indicating that microclimate data should be used for indoor thermal comfort assessment and energy performance, but encountering that the passive mode appeared not viable, the addition of the biomimetic strategies in [36], which improved the microclimate conditions, could increase the viability of the use of natural ventilation, and the buildings’ energy performance in this urban zone.

That is why the use of the same biomimetic strategies in [36] are applied to the base case study (namely, proposed case), to assess the thermal and energy performance of the urban zone. The performance is evaluated by means of indoor thermal comfort indicators, such as operating temperature, surface temperature, as well as the evaluation of the satisfied hours of natural ventilation (SHNV), using dynamic simulation through the Designbuilder software v6.1.6.011 [37] and ENVI-met. v4.4.5 [38]. The evaluation was carried out in March (driest) and October (rainiest), comparing the base case (or reference case) and the proposed case. Besides, full reflective coating is applied to the base case and compared with both other cases in terms of performance. Since the combined biomimetic strategy of [36] surpasses in performance the full reflective coating approach, a 2D transient heat transfer model is constructed to clarify this outcome contrasted with those in [25].

## 2. Materials and Methods

**Figure 1:**
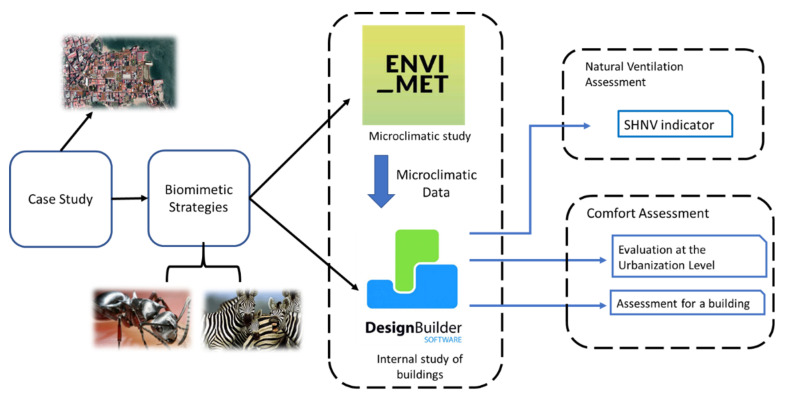
Proposed methodology for the development of the research. Own elaboration.

For the development of this research, the methodology implemented consists of evaluating microclimatic data within an urban zone in the Old Town of Panama City named Casco Antiguo via ENVI-met and the corresponding standard typical meteorological data. The microclimatic data were adapted via a numerical approach to assessing the energy performance of buildings within the urban zone [8]. The Designbuilder software, together with the microclimatic data, were used to assess such performance of buildings operative in passive and active modes for both the base case and the proposed case. The proposed case is based on biomimicry strategies (Figure 1).

### 2.1. Case Study and Problem Identification

**Figure 2:**
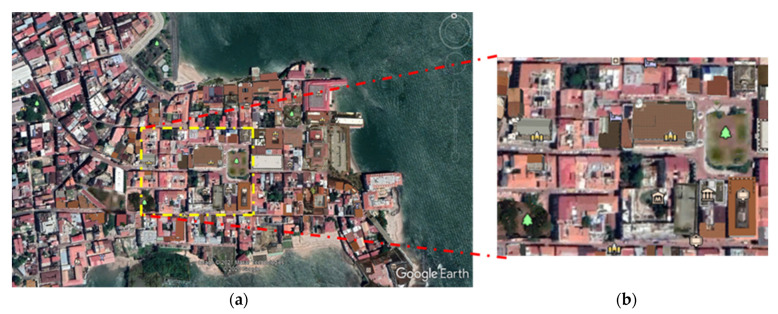
Urban zone under study: (**a**) Satellite view of the Casco Antiguo, and (**b**) cutout view of (290 m × 226 m) [36].

The study focuses on the Old Town of Panama City, Caso Antiguo, which is declared a World Heritage Site by UNESCO. Its colonial design is based on the laws of the Indies, in which its buildings are mainly composed of materials such as: wood, calicanto, clay blocks, concrete, tile roofs, and concrete slab roofs [1]. The area is classified as a climatic zone type 3 (LCZ3) according to Oke [39], which consists of high-density, low-level buildings (3 to 4 levels), paved ground, and few trees. To perform the numerical study, part of this urban zone was analyzed: the cutout chosen covers a dimension of 290 m (x) by 226 m (y), as can be seen in Figure 2. This urban zone was selected to analyze further previous studies conducted for the same cutout [1,8,36,40]. Table 1 summarizes the materials of the buildings in the studied zone or cutout.

**Figure 3:**
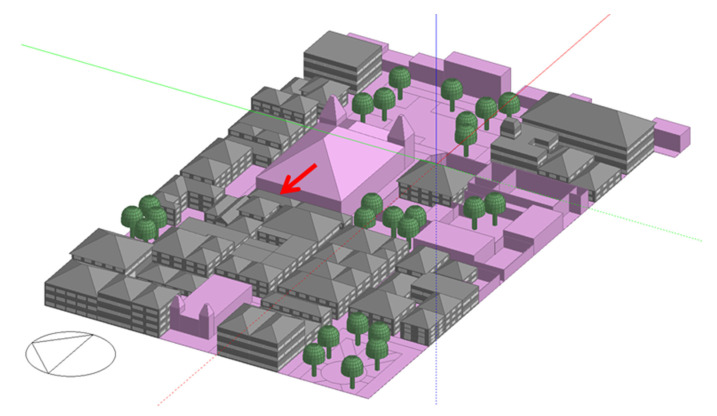
Base case 3D model in Designbuilder. Own elaboration.

Figure 3 shows the 3D model of the base case study area in Designbuilder and Table 2 presents the transmittance values for each of the buildings’ envelope elements. Using the Google Earth Pro tool, the dimensions of the streets and buildings were taken from the cutout, and the Designbuilder blocks tool was used to create the buildings. Only the energy consumption and thermal comfort of gray-colored buildings are considered in the simulation results. The uninhabited buildings, churches, and historical monuments are shown in color pink, which indicates that their individual energy consumption and thermal comfort are not considered in the simulation results but their impact on other buildings performance is considered, through shading, wind blocking, and reflectiveness. In addition to the information related to the heights of the buildings, the construction materials according to the real characteristics of the pavements, surfaces, and vegetation (only trees without the consideration physiological functions, such as evapotranspiration) were placed.

### 2.2. Proposed Designs and Simulation

An evaluation of the study area previously proposed, in which applied biomimetic strategies focused on a problem-based approach, i.e., the basis of an exhaustive search for biological analogies, where the most related were extracted thanks to the biomimetic design methodology [36]. Such biological analogies were applied on the buildings’ roofs to evaluate their effects on pedestrians’ comfort. Now, the present study concentrates on evaluating their effects on indoor thermal comfort and energy consumption.

#### 2.2.1. Abstraction and Emulation of the Identified Biomimicry-Based Strategies

**Figure 4:**
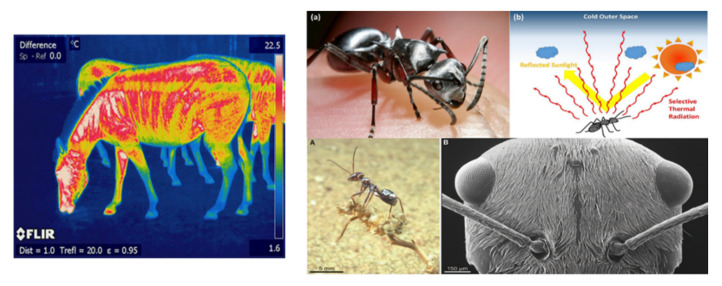
Convection currents due to temperature variation in the black and white lines. Infrared photo with the respective surface temperature variations (**left**). Characteristics of the Saharan ant (**right**) ([41,43]).

The biomimicry problem-based approach applied to the case study led to abstract and emulated a two-pinnacle strategy based on the Zebra stripes and the Saharan ant. The Saharan ant for its highly reflective characteristics, and the zebra specifically its black and white appearance, to emulate convective currents and achieve evaporative cooling (Figure 4). Table 3 shows the summary of such pinnacles’ analysis [36].

**Figure 5:**
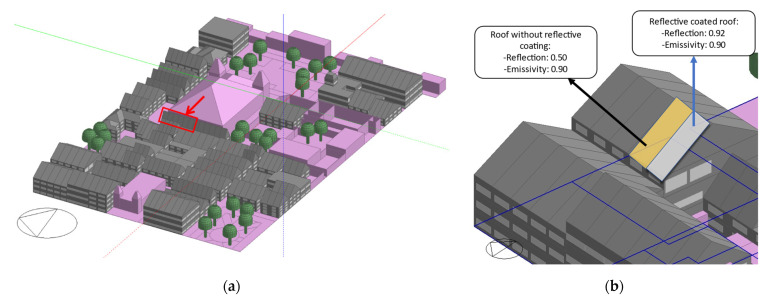
Proposed case: (**a**) 3D model in Designbuilder (selected building for further analysis inside red box) and (**b**) the order of applied reflectivity and its properties. Own elaboration.

The emulation of such pinnacle strategies was implemented in the urban zone, as in [36] but in the Designbuilder software on the buildings’ roofs. For this, an additional layer of a reflective coating was added to the based-case pitched roof construction (Table 4), following the Zebra-stripes pattern interchanging between the reflective coating and the original roof, as shown in Figure 4. A change in the pitched roof geometry was made from a multi-slopes roof to a two-slope roof for simplicity to adjust and include the stripes pattern. The reflective lines added are 1 cm thick and the width depended on the length of each building ranging from 4 to 6 m wide; no uniform stripe width and shape may be key for Zebras [42]. The buildings’ roofs with flat roof constructions were kept as in the base case. The 3D model of the proposed case developed in Designbuilder is shown in Figure 5. To evaluate the base case further, the reflective coating was added to all pitched roof constructions (on gray-colored buildings) for comparison purposes to the proposed case. The value of the coating was based on the value of the reflective properties of the Saharan ant 0.97 [43], commercially a high value of reflective coating of 0.92 [44] was found and therefore that value was taken which was the closest.

#### 2.2.2. Simulation of the Based and Proposed Cases

**Figure 6:**
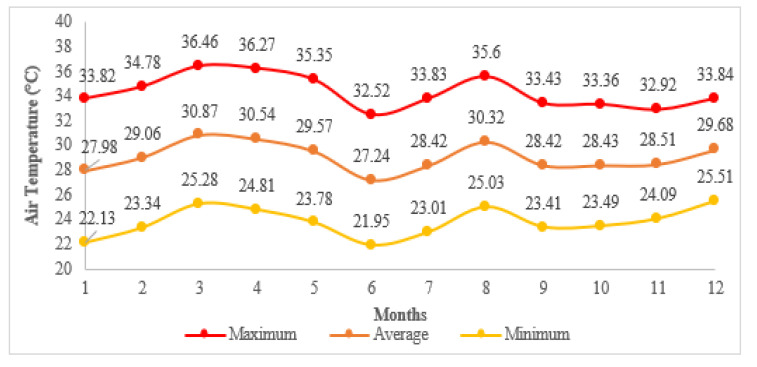
Resulting air temperature of the microclimate data from ENVI-met simulations [8].

The simulations for both cases were performed using microclimatic data extracted from ENVI-met for the whole year. These microclimatic data were created using a numerical approach as in [8] and results from simulations carried out in ENVI-met using standard weather data (typical meteorological data) obtained from CLIMdata Solargis © (Table 5). The resulting microclimatic data for the air temperature is presented in Figure 6.

The main activity in the Casco Antiguo is commercial, such as restaurants and bars, equivalent to 50% of the buildings, another 40% is dedicated to hotels and residences, and 10% are offices and public institutions. The cut-out of the old town has a different occupation profile from another area of the city [8].

**Figure 7:**
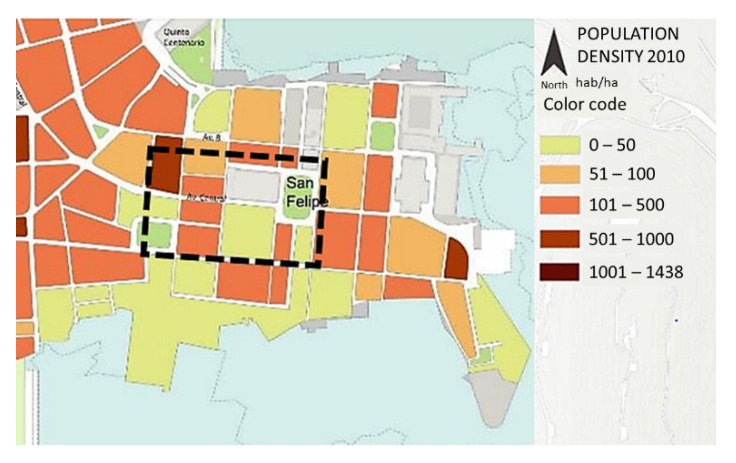
Population density in the urban zone (adapted from [8]). The studied zone inside black dashed square.

The town of San Felipe has a population of approximately 3262 people and is where the Casco Antiguo is located. The population density is presented in [8] (Figure 7), which was considered equivalent to the occupation profile carried out in the simulation (Table 6).

### 2.3. Buildings’ Performance Evaluation and Comparison

The buildings’ performance was evaluated in terms of the following indicators: indoor thermal comfort, roof heat gains, and the electricity consumption for cooling. This evaluation was performed at the building level, i.e., the average values for the entire studied urban zone. The resulting values for each indicator were analyzed and compared in monthly and hourly averages, depending on the indicator. The urban zone was subjected to two operation modes: passive and active modes. The latter mode corresponds to an operation under the use of air conditioning, and the former mode corresponds to an operation under natural ventilation only. This was completed beforehand for both the base case and proposed case.

#### 2.3.1. Passive Mode Operation

The building thermal performance during passive operation mode was assessed using the following indicators: Operative temperature, roof heat gains, and the Satisfied hours of natural ventilation (SHNV). Results are obtained only for occupied periods for monthly indicators. The SHNV is introduced here as it assesses the possibility of ensuring acceptable indoor air quality and natural thermal comfort. The latter corresponds to the percentage of the maximum number of hours when the outdoor climate is favorable for natural ventilation inside buildings, based on the thermal comfort requirements established in the ASHRAE 55 standard for naturally conditioned areas [45]. This indicator can be significantly affected by the outdoor climate, effects of building characteristics such as design, internal heat gains, and envelope energy performance. Finally, the criteria for the SHNV indicator are defined based on the average outdoor air temperature and the operative temperature, as the intersection of these two temperatures for an acceptable 80% ambient. If the thermal comfort condition does not meet the established criteria, this indicator equals zero.

#### 2.3.2. Active Mode Operation

The building thermal performance during active operation mode was assessed by focusing on the electricity consumption for cooling purposes, only for occupied periods. The cooling temperature was set to 24 °C considering air conditioning devices with a coefficient of performance of 3.00. Natural ventilation was permitted during unoccupied periods.

#### 2.3.3. Heat Transfer Analysis of the Zebra-Stripe Strategy

**Figure 8:**
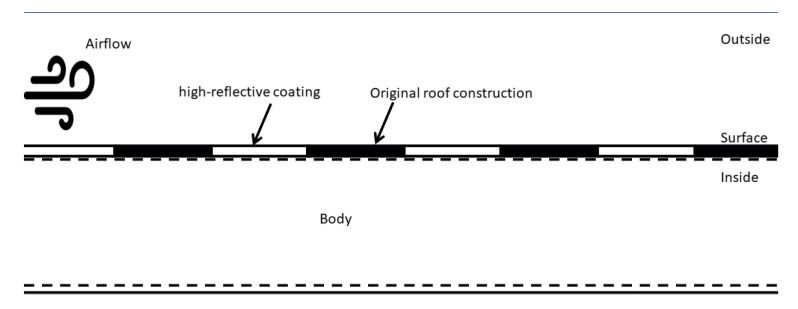
The 2D heat transfer transient setup to evaluate the contrasted stripes strategy. Own elaboration.

Other studies concerning the Zebra striped skin have associated this strategy with the survival way to regulate skin and body temperature. Experimental studies evidenced a significant temperature difference between white and black stripes. Thus, in order to further understand the building energy performance simulation results and for complementary purposes, 2D heat transfer transient simulations were carried out via the Energy2D software [46] based on Designbuilder results. The 2D heat transfer model setup is presented in Figure 8.

To simplify the transient heat transfer simulation and since the Energy2D software solves the heat conduction equation together with the conservation laws, the following conditions were considered:

1. The thermal boundary conditions for the outside were considered as the same as the air temperature; this was set to 0 °C (default temperature value for any surrounding in the software), except for the bottom boundary, which was set to the same temperature as the body. The mass boundaries were set as open. The thermal properties corresponded to the set-up temperature;
2. The body may represent the body of the Zebra or the roof construction material just below the coated surface. Its temperature was set to 50 °C throughout the entire simulation;
3. The outside airflow speed remained constant at 0.01 m/s (using a default ventilator). The time step was set to 10 s, and the simulation duration was limited to 30 min;
4. The stripes were also kept at a constant temperature value. The original roof layer was set to the same temperature as the body, and the high-reflective coating was set to 10 °C;
5. The values for the thermal conductivity, specific heat capacity, and density remained as the default values: 1 W/mK, 1300 J/kgK, and 25 kg/m^3^;
6. Any radiation heat transfer interaction was neglected since the stripes’ temperatures were set at a constant value, to consider it in advance.

This setup does not intend to replicate the exact situation considered for the energy performance simulations, but it may serve as a reference setup to evidence and understand the heat transfer problem. Finally, since a finite-difference method is used to solve the equations, all physical contact among the stripes and body are considered.

## 3. Results Analysis

This section presents the analysis of the results and their discussion, starting with evaluating the indoor thermal performance via operative temperature and SHNV levels. A brief analysis of the thermal lag and damping factor results is presented. This is followed by the energy efficiency evaluation in terms of roof surface temperatures and heat gains and the electricity consumption for cooling. Finally, the 2D transient heat transfer simulation results are presented.

### 3.1. Evaluation of the Thermal Performance via Operative Temperature and SHNV

**Figure 9:**
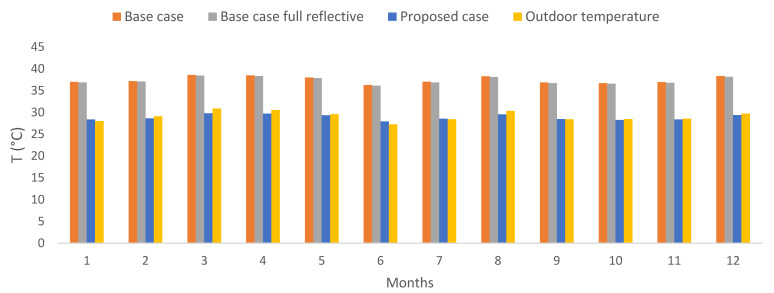
Monthly results of the operative temperature for the urban zone, only for occupied hours. Own elaboration.

The average operative temperature results for the entire urban zone can be seen in Figure 9. It can be clearly seen that the proposed case presented a significant reduction concerning the base case, reaching 8.51 °C of reduction for March (from 38.28 °C to 29.77 °C) after applying the Zebra-stripes strategies. The same happened in October with a 9.59 °C reduction. This reduction presented an average of approximately 8.82 °C (from 37.66 °C to 28.84 °C) throughout the year. The main reason for this reduction is associated with the application of the reflective layer, which prevents a large part of the solar radiation that falls on the building from penetrating inside it, which makes the operative temperature lower. Results also showed that when implementing the reflective coating for all pitched roofs in the base case, the monthly operative temperature values obtained are higher than in the proposed case.

**Figure 10:**
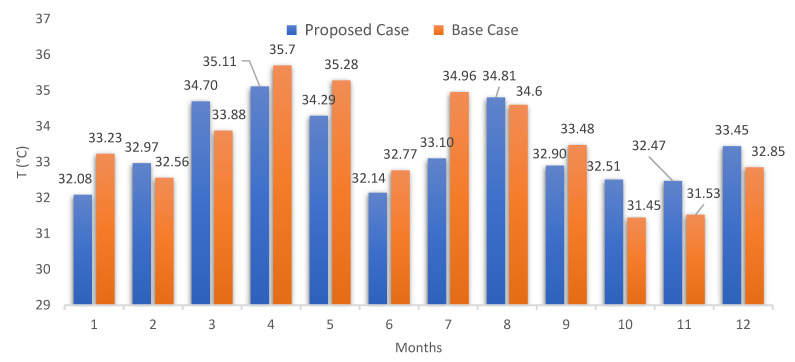
Monthly results of the operative temperature for the selected building, only for occupied hours. Own elaboration.

Subsequently, an inspection throughout the urban zone allowed noticing that only a two-story building presented acceptable values for the SHNV calculation (selected building in red framed in Figure 5); the same building as the base case. Note here that Figure 9 shows the operative temperature results for the entire urban zone, while Figure 10 shows the operative temperature for the selected building. Temperature reductions can be observed for six months.

Accounting only for the selected building, the results of the operative temperature for the critical day of each month were analyzed in detail, assuming this behavior for the rest of the month: 20 February, 20 October, and 11 November. These dates were chosen because they presented acceptable values for the SHNV in the base case. Hourly temperature results indicate that the maximum temperature is no longer reached as specified by the standard weather data (Table 4); maximum values are reached at 16 h and 17 h for outdoor air temperature and operative temperature, respectively. This evidences the impact of the use of microclimatic data. However, for the proposed case with these operative temperature results and the already recorded outdoor air temperature values (Figure 6), only October presented acceptable values for the evaluation of the SHNV. The corresponding SHNV for October resulted in 37.5% (279 h of the 744 h), which conduce to a 3.18% for the entire year. For the base case, the SHNV for February, October, and November resulted in 4.17% (28 h), 16.67% (124 h), and 16.67% (120 h), respectively, conducing to 3.11% for the entire year. These results confirm the thermal unconformity recorded inside the buildings during the occupancy period of the buildings in passive mode, and such values are considered significantly low according to Causone in [47].

Moreover, two parameters that indicate the envelope’s thermal performance were evaluated between the proposed case and the base case shown in the table. Such is the thermal lag, which refers to the time it takes to transfer energy in the form of heat from the exterior to the interior in hours, and the damping, which is a fraction between the difference in max and min temperatures of the interior and exterior of the building [48]. In Table 7, it can be observed that according to the thermal lag in November, the operative temperature reached its peak value (or maximum value) faster in the proposed case than in the base case. No significant changes were observed for other months. Therefore, there is a greater heat transfer penetration in the proposed case than in the base case.

### 3.2. External Surface Temperature for the Proposed Case

**Figure 11:**
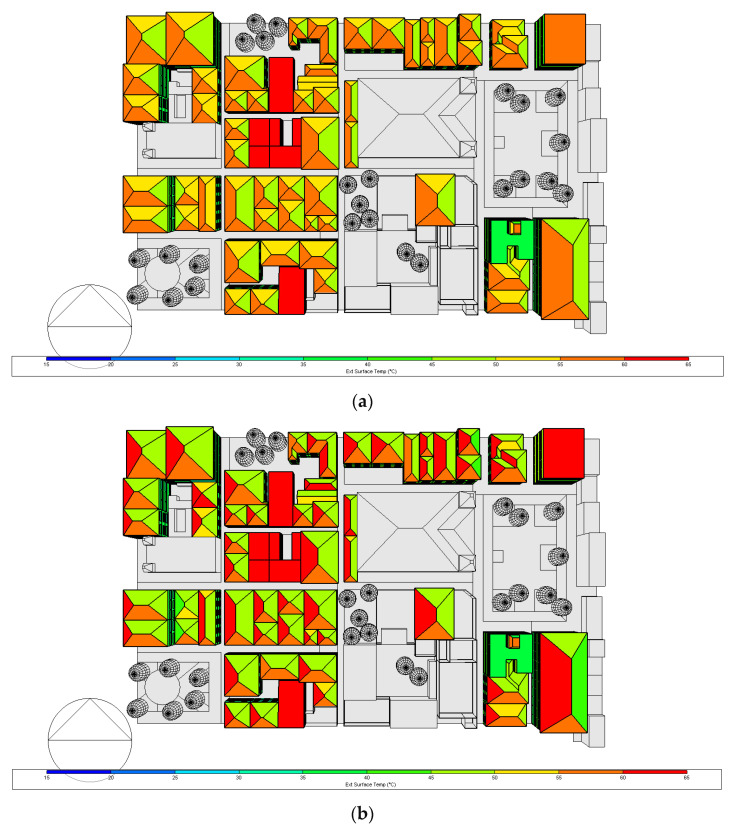
External surface temperature for the base case: (**a**) 17 March and (**b**) 20 October at 15 h. Own elaboration.

**Figure 12:**
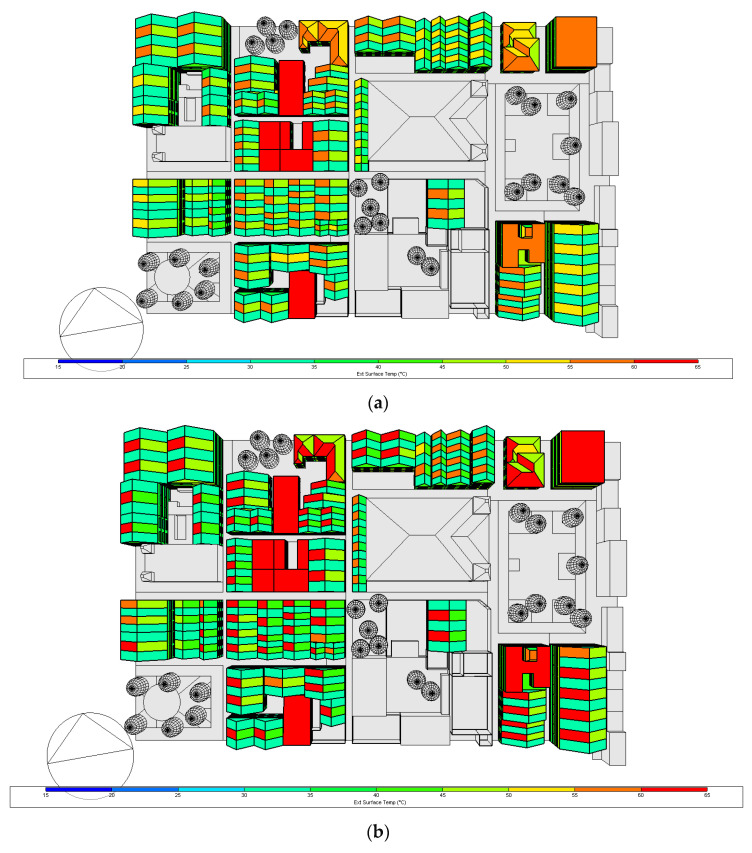
External surface temperature for the proposed case: (**a**) 17th March and (**b**) 20th October at 15 h. Own elaboration.

The external surface temperature results for 17 March and 20 October, at 15 h, are presented in Figure 11 and Figure 12, for the base case and proposed case, respectively. As it is 3:00 p.m., it can be seen that the temperature is higher on the west side of the roofs, in March the vast majority of the roofs oscillated 55–60 °C and the east side 45–50 °C, by October the west side of the roof oscillates between 60–65 °C and the east side between 45–50 °C—therefore for the base case in October a higher temperature is obtained.

As expected, significant differences between the reflective coating and the original roof construction surface temperature can be observed. Similarly, low surface temperature values were obtained when implementing the reflective coating for all pitched roofs in the base case. Regarding the monthly average surface temperature values, for March, the difference between the roof of the same building using the Zebra-stripes biomimetic strategy (ZSBS) oscillates in a range of 27.5 °C and 30 °C in the reflective segment and in the non-reflective segment between 32.5 °C and 35 °C, that is, a difference of 5 °C between both segments. For flat buildings in which the ZSBS were not applied, the temperature difference of these buildings concerning those with strategies is about 5 °C.

For October, the difference between the roof of the same building having the ZSBS oscillates in a range of 25–27.5 °C in the reflective segment and in the non-reflective segment between 32.5 °C and 35 °C, corresponding to a difference of 7.5 °C between both segments. In buildings not having the ZSBS, the temperature difference concerning those that have the ZSBS is 3.75 °C. For March, considering the complete area of the model, the proposed case decreased 7.5 °C with respect to the base case. The same happened for October.

### 3.3. Comparison of Roof-Ceiling Heat Gains and Cooling Electricity Consumption

**Figure 13:**
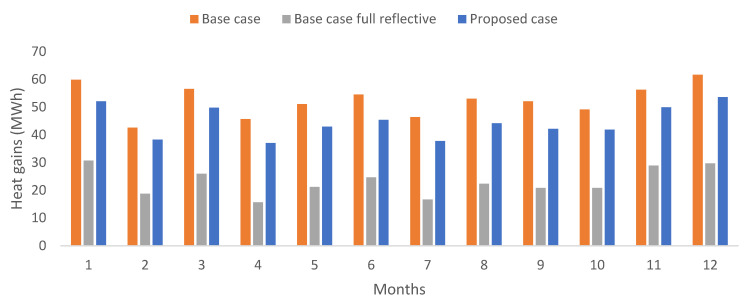
Comparison of monthly roof-ceiling heat gains for the urban zone, during occupied hours. Own elaboration.

Figure 13 shows the monthly averages for the roof-ceiling heat gains for the urban zone during the occupied hours for the base and proposed cases. Besides, the base case when implementing the reflective coating on all pitched roof constructions is shown. As expected, the lowest heat gains are encountered for the base case with fully reflective coating on all pitched roofs. The proposed case presented slight reductions concerning the base case.

**Figure 14:**
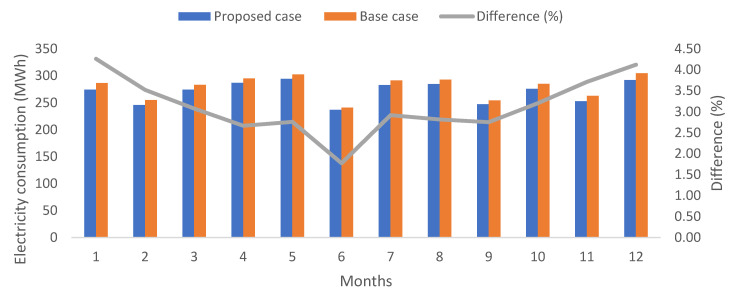
Comparison of monthly electricity consumption for cooling (only for occupied hours). Own elaboration.

On the other hand, the electricity consumption for cooling needs can be observed in Figure 14. Slight differences are encountered between the base and proposed cases, with an average difference of 3.13%. The latter corresponds to an annual reduction of 8790 kWh, which is significant when considering electricity costs, but this should be addressed further contrasted with the retrofit costs to implement the proposed case at a real scale. The active operation mode was not simulated when implementing the reflective coating on all pitched roofs for the base case.

### 3.4. Heat Transfer Analysis of the Zebra-Stripes Strategy

**Figure 15:**
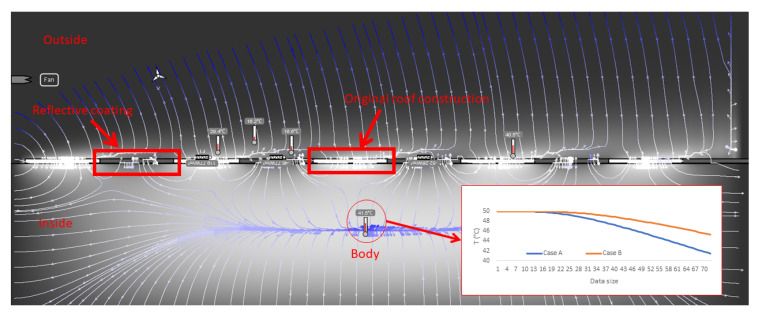
Transient simulation results for the 2D heat transfer model with and without segments reflective coating. Case A: Zebra-stripes strategy and Case B: reflective coating covered the entire surface. Own elaboration.

Considering that the proposed case has presented significant results with respect to the base case and base case with fully reflective pitched roof construction. The 2D transient heat transfer simulation results when implementing the ZSBS are presented in Figure 15 (case A). Gray color flashes indicate the temperature range where the highest temperature zones (50 °C) correspond to the clearer zones (in white) and the lowest temperature zones (0 °C) to the darkest. The blue lines represent the heat flux lines. These results were compared with the case where the reflective coating covered the entire surface (case B) in terms of a temperature measurement at the center of the “body”. At the end of the simulation time, this temperature measurement reported a lower value for case A than case B, with a difference of 3.8 °C. The graph in Figure 15 shows that in the one with total reflectivity (case B, orange line), the temperature decreases more slowly than the one with the biomimetic strategy (case A, blue line). In more detail, case A shows distortions of the heat flow lines around the contrasting segments, which indicates that the temperature differential in the segments increases heat dissipation, explaining its speed compared to case B.

## 4. Discussion

A proposed model was made based on the emulation of the characteristics of the Saharan ant employing a high reflectivity coating applied to the buildings’ roofs and the contrasted stripes of the Zebra by applying the reflective coating in a segmented distribution. All this is using the Designbuilder software in which the microclimatic data were taken for the dynamic simulation carried out in the ENVI-met software. From the simulation results obtained, the following could be highlighted:

- In the proposed case, the indoor operative temperature of the entire urban zone under study was reduced by about 8–10 °C on average for the year, compared to the base case, due to the application of the reflective coating on the roof, preventing heat gain in inside;
- Regarding the external surface temperature of a building with biomimetic strategies for March, the reflective and non-reflective segments presented a temperature difference of about 5 °C, and it was also found that the building with strategies is 5 °C colder than a building without the strategies. In October, the temperature difference between the reflective and non-reflective segments was 7.5 °C, while buildings with strategies have 3.75 °C less (colder) without applied strategies;
- The interior temperature for damping for November in the proposed case reached its peak value faster than the base case due to a reduction in the hours of the thermal lag indicator;
- Significant reduction in the electricity consumption for cooling was achieved by the proposed case, with respect to the base case, with an average of 3.13% (about 8.8 MWh);
- The simplified setup for 2D transient heat transfer simulations evidenced enhanced heat transfer dissipation for the proposed case compared to a fully reflective coating on the surface.

Although the operative temperatures reached lower values than the base case, results from the SHNV indicator confirm the thermal unconformity recorded inside the buildings during the occupancy periods in passive mode, which is considered significantly low just as in [47].

Moreover, the proposed case performed better than the base case and the base case when implementing the reflective coating over all roof constructions in terms of indoor operative temperature values and electricity consumption for cooling. The average reduction in electricity consumption for cooling of 5.07% (about 505 kWh) when using microclimate data as in [8], compared to the average reduction of 3.13% (8.8 MWh), here in the proposed case, might not represent high enough energy savings to undergo the microclimate data generation process from standard weather data or in situ data collection for simulation purposes only, as suggested in [8,49]. However, the use of microclimate data could strengthen the evaluation-verification process of new urban designs [49] before implementation at a real scale by giving more precise values for energy needs forecast.

Furthermore, the difference in the roofs with the applied Zebra-stripes biomimetic strategy is comparable as it occurs with the Zebra, which through its black and white coloration, obtains a temperature differential that causes convective currents that accelerate heat dissipation [26]. The effectiveness of this biomimetic strategy is also observed at the surface temperature at the roof west slopes of both cases for both months (Figure 11 and Figure 12). For instance, above the park on the left (surrounding with trees), the buildings’ roofs west slope without the reflective coating (color yellow in Figure 12a) appeared 5 °C lower than the same slopes in Figure 11a (in orange). The same can be spotted on the buildings’ roofs above the cathedral and those on the righthand side (below the park).

To corroborate, the 2D transient heat transfer simulations helped realize that the heat flow perturbance at the surface of the body (heat flow lines in Figure 15) caused by the stripes allows a faster temperature drop than the surface with homogenous heat flow. However, the impact of this strategy could be enhanced by combining different patterns since the latter may be as fundamental as the stripes’ pattern distribution and shape [25]. It is worth highlighting, based on the study [25] that denies the fact that Zebra stripes do not have a significant effect on the decrease in body temperature, our study is based on biomimetics, which does not take the behavior of the analogy directly. Rather, it is adapted to the applications and needs of the design, for example, the reflective coating that also promotes heat dissipation. Finally, using a commercial coating with a reflectivity value of 0.92 [44] on roofs limits the Saharan ant reflectivity value of 0.96, which brings an opportunity for future coating developments. Although significant energy saving for cooling was attained with the combined biomimetic strategy used here, a cost-effective analysis of retrofitting the roofs’ construction contrasted with energy saving costs, needs to be performed, as in as in [21], before implementation.

## 5. Conclusions

The main objective of this research was to evaluate the indoor thermal comfort and energy efficiency of buildings in an urban area in the Casco Antiguo of Panama City through biomimetic strategies from a previously carried out study. Among the findings this investigation provided, the following can be retained: The functionality of Zebra stripes, together with the reflective characteristics of the Saharan ant, provide better performance for buildings’ thermal regulation and energy needs for cooling. Further investigation through experimental studies might support the effectiveness of this combined biomimetic strategy, but a cost-effective analysis of retrofitting the roofs’ construction contrasted with costs energy saving needs to be performed before implementation.

It is important to highlight that biomimetics has been applied throughout history by nature without generating negative impacts on the environment. That is why it must be learned from, and we must seek alternatives to solve the challenges currently faced by society through the application of different approaches so that they may be solved sustainably.

A recommendation for future work would be to conduct more in-depth studies verifying the effectiveness of the strategies applied in this study, such as convective currents that influence heat dissipation through experimental tests. The evaluation of the application of biomimetic strategies in walls added to the strategies of this study.

## Figures and Tables

**Table 1:** Characteristics of materials employed in the 3D model [8].

Material	Texture	Conductivity (W/mK)	Specific Heat (J/kgK)	Density (kg/m^3^)
Concrete block	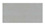	1.04	921.10	1841.10
Clay tile	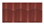	1.00	800	2000
Brick pavement	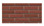	0.96	840	2000
Concrete pavement	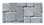	0.96	840	2000
Cultivated clay soil	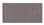	1.18	1250	1800

**Table 2:** Characteristics of materials employed in the 3D model. Own elaboration.

Building Envelope Element	Transmittance Values (W/m^2^K)
External walls	3.087
Internal partitions	1.639
Pitched roof (base case)	2.930
Flat roof	0.250
Pitched roof (proposed case) with reflective layer	2.828
Windows	5.778
Ground floor	0.350
Internal floor	2.929

**Table 3:** Summary of the Pinnacles Analysis. Own elaboration.

Pinnacles Strategy	Mechanism	Fundamental Principles	Main Feature
Zebra	Black and white streaking causes a temperature differential [30,41].	Convective currents are caused by increasing evaporation.	High convection and evaporation
Arrangement of animal stripes for heat regulation			
Saharan ant	High reflection in the NIR range and emissivity in the NIR [17].	Reflection of thermal radiation and high emissivity to release excess heat	High reflectivity and emissivity
Silver hairs with triangular structure			

**Table 4:** Layers in the pitched roofs based on the base case. Own elaboration.

Material	Thickness (m)	Reflectivity (-)
Reflective Coating	0.010	0.92
Clay Tile (Roofing)	0.0250	0.3
Air Gap	0.0200	-
Roofing Felt	0.0050	0.2

**Table 5:** Standard weather data used for simulation. Own elaboration.

Month	Tmax (°C) (Hour)	Tmin (°C) (Hour)	HRmax (%) (Hour)	HRmin (%) (Hour)	Averaged Wind Speed (m/s)	Averaged Wind Direction (°)
3 January	35 (15:00)	23.9 (6:00)	94 (5:00)	44 (15:00)	0.43	126
20 February	34.6 (15:00)	22.2 (6:00)	93 (6:00)	40 (15:00)	2.77	85.77
17 March	35.6 (15:00)	24.9 (6:00)	73 (6:00)	36 (16:00)	2.3	49
11 April	35.3 (15:00)	24.8 (6:00)	82 (24:00)	44 (16:00)	1.75	87
20 May	34.8 (15:00)	24.5 (6:00)	90 (6:00)	53 (16:00)	0.87	83.3
23 June	32.8 (15:00)	23.4 (6:00)	94 (6:00)	58 (15:00)	0.45	108.25
21 July	35.5 (16:00)	24.3 (6:00)	97 (4:00)	49 (16:00)	0.3	89.3
19 August	34.7 (15:00)	24.1 (6:00)	95 (5:00)	52 (15:00)	3.9	188
1 September	32.5 (15:00)	23 (6:00)	98 (24:00)	60 (15:00)	2.1	83
20 October	32.5 (15:00)	23 (6:00)	96 (6:00)	62 (14:00)	2.33	90.67
11 November	32.9 (15:00)	23.7 (6:00)	94 (5:00)	61 (13:00)	2.55	80
16 December	34.3 (15:00)	24.6 (6:00)	94 (7:00)	50 (16:00)	4.2	34.5

**Table 6:** Occupied periods and energy usages in the urban zone [8].

Occupancy and Energy Usages	Schedule
Occupation Profile: 0–0.005 hab/m^2^ 0.0051–0.01 hab/m^2^ 0.0101–0.05 hab/m^2^ 0.0501–0.1 hab/m^2^	Monday to Friday: 8:00 to 18:00 Saturday to Sundar: 13:00 to 17:00
Luminaires (24 W)	Monday to Friday: 19:00 to 22:00 Satday to Sunday: 19:00 to 5:00
Fans (70 W)	Monday to Friday: 12:00 to 16:00 Sat to Sun: 11:00 to 16:00
Computers (65 W)	Mon to Fri: 9:00 to 17:00
Refrigerator (145 W)	Sun to Sat: 0:00 to 23:59
Air conditioning unit	Mon to Fri: 9:00 to 17:00 Satday a Sunday: 10:00 a 22:00

**Table 7:** Comparison of the thermal lag and damping factor, for both cases. Own elaboration.

	Date	Thermal Lag (h)	Damping Factor (-)
Base case	20 February	1	1.0046
	20 October	1	0.9562
	11 November	4	0.8648
Proposed case	20 February	1	0.9799
	20 October	1	0.9805
	11 November	3	0.8745

## Data Availability

Not Applicable.

## References

[1] Collado A., Bustos Romero M.A. (2021). Microclimas urbanos en la Ciudad de Panamá: Estudio de tres recortes históricos de la ocupación urbana. Paranoá Cad. Arquitetura Urban..

[2] Las Ciudades Seguirán Creciendo, Sobre Todo en los Países en Desarrollo|ONU DAES|Naciones Unidas Departamento de Asuntos Económicos y Sociales. https://www.un.org/development/desa/es/news/population/2018-world-urbanization-prospects.html.

[3] Pérez H., Flores J., López A. Modelo de Ventilación Inducida Para la Vivienda en Clima Cálido Húmedo: Sistema Chimenea Solar. Forum Latinoamericano de Engenharia. Eng. Foz de Iguaçu, Brazil, 11–13 November 2013. https://flaequnila.wixsite.com/flaeq/i-flaeq.

[4] (2016). Secretaría Nacional de Energía Guía de construcción sostenible para el ahorro de energía en edificaciones (RES). Gac. 24 noviembre 2016.

[5] Oficial G. Plan Energético Nacional 2015–2050. 2016, pp. 6–348. http://www.energia.gob.pa/plan-energetico-nacional/.

[6] Villarreal D., Candanedo M. (2020). Efecto de las islas de calor urbano en las principales vías de la Ciudad de Panamá. I+D Tecnológico.

[7] Austin M.C., Garzola D., Delgado N., Jiménez J.U., Mora D. (2020). Inspection of Biomimicry Approaches as an Alternative to Address Climate-Related Energy Building Challenges: A Framework for Application in Panama. Biomimetics.

[8] Rodríguez Maure K., Mora D., Chen Austin M. (2021). Buildings Energy Consumption and Thermal Comfort Assessment Using Weather and Microclimate Data: A Numerical Approach In Humid-Tropical Climate. Proceedings of the 19th LACCEI International Multi-Conference for Engineering, Education and Technology, Virtual.

[9] What Is Biomimicry?—Biomimicry Institute. https://biomimicry.org/what-is-biomimicry/.

[10] Fu S.C., Zhong X.L., Zhang Y., Lai T.W., Chan K.C., Lee K.Y., Chao C.Y.H. (2020). Bio-inspired cooling technologies and the applications in buildings. Energy Build..

[11] Durai Prabhakaran R.T., Spear M.J., Curling S., Wootton-Beard P., Jones P., Donnison I., Ormondroyd G.A. (2019). Plants and architecture: The role of biology and biomimetics in materials development for buildings. Intell. Build. Int..

[12] Radwan G.A.N., Osama N. (2016). Biomimicry, an Approach, for Energy Effecient Building Skin Design. Procedia Environ. Sci..

[13] Badarnah L., Nachman Farchi Y., Knaack U. (2010). Solutions from nature for building envelope thermoregulation. WIT Trans. Ecol. Environ..

[14] Hair Helps Cool the Body—Biological Strategy—AskNature. https://asknature.org/strategy/hair-helps-cool-the-body/.

[15] Head Position Helps Correct for Light Refraction—Biological Strategy—AskNature. https://asknature.org/strategy/head-position-helps-correct-for-light-refraction/.

[16] Shell Protects From Heat—Biological Strategy—AskNature. https://asknature.org/strategy/shell-protects-from-heat/.

[17] Shi N.N., Tsai C.C., Camino F., Bernard G.D., Yu N., Wehner R. (2015). Keeping cool: Enhanced optical reflection and radiative heat dissipation in Saharan silver ants. Science.

[18] Schmidt-Nielsen K., Taylor C.R., Shkolnik A. (1971). Desert snails: Problems of heat, water and food. J. Exp. Biol..

[19] Tsoka S. (2020). Influence of Aging on the Performance of Cool Coatings.

[20] Wang J., Liu S., Meng X., Gao W., Yuan J. (2021). Application of retro-reflective materials in urban buildings: A comprehensive review. Energy Build..

[21] Pomerantz M. (2018). Are cooler surfaces a cost-effect mitigation of urban heat islands?. Urban Clim..

[22] Santamouris M., Synnefa A., Karlessi T. (2011). Using advanced cool materials in the urban built environment to mitigate heat islands and improve thermal comfort conditions. Sol. Energy.

[23] Lei J., Kumarasamy K., Zingre K.T., Yang J., Wan M.P., Yang E.H. (2017). Cool colored coating and phase change materials as complementary cooling strategies for building cooling load reduction in tropics. Appl. Energy.

[24] Shanmuganathan R., Sekar M., Praveenkumar T.R., Pugazhendhi A., Brindhadevi K. (2021). Experimental investigation and numerical analysis of energy efficiency building using phase changing material coupled with reflective coating. Int. J. Energy Res..

[25] Horváth G., Pereszlényi Á., Száz D., Barta A., Jánosi I.M., Gerics B., Åkesson S. (2018). Experimental evidence that stripes do not cool zebras. Sci. Rep..

[26] Cobb A., Cobb S. (2019). Do zebra stripes influence thermoregulation?. J. Nat. Hist..

[27] Webb M. (2021). Biomimetic building facades demonstrate potential to reduce energy consumption for different building typologies in different climate zones. Clean Technol. Environ. Policy.

[28] Imani N., Vale B. (2020). A framework for finding inspiration in nature: Biomimetic energy efficient building design. Energy Build..

[29] Elliot T., Rugani B., Almenar J.B., Niza S. (2018). A Proposal to Integrate System Dynamics and Carbon Metabolism for Urban Planning. Procedia CIRP.

[30] Martín-Gómez C., Zuazua-Ros A., Bermejo-Busto J., Baquero E., Miranda R., Sanz C. (2019). Potential strategies offered by animals to implement in buildings’ energy performance: Theory and practice. Front. Archit. Res..

[31] Fecheyr-Lippens D., Bhiwapurkar P. (2017). Applying biomimicry to design building envelopes that lower energy consumption in a hot-humid climate. Archit. Sci. Rev..

[32] Marlén Lòpez M. Envolventes Arquitectónicas Vivas Que Interactúan Con Su Entorno-Naturalizando El Diseño. Ph.D. Thesis, Departamento de Construcción e Ingeniería de Fabricación, Universidad de Oviedo, España.. https://digibuo.uniovi.es/dspace/bitstream/handle/10651/45074/TD_marlenlopez.pdf?sequence=6&isAllowed=y.

[33] Sari D.P. (2021). A Review of How Building Mitigates the Urban Heat Island in Indonesia and Tropical Cities. Earth.

[34] Architectureever Lavasa Township|It’s Bio-Mimetic history|Biomimicry|India|Architecturever. https://architecturever.com/2019/04/08/lavasa-township-and-its-bio-mimetic-history/.

[35] Brown J.D. Singapore Summit—Biophilic Cities 2019. https://www.biophiliccities.org/singapore-summit-reflections.

[36] Araque K., Palacios P., Mora D., Chen Austin M. (2021). Biomimicry-Based Strategies for Urban Heat Island Mitigation: A Numerical Case Study under Tropical Climate. Biomimetics.

[37] DesignBuilder Software Ltd DesignBuilder 2018. http://www.designbuilder.co.uk/.

[38] Home—ENVI-Met. https://www.envi-met.com/es/.

[39] Stewart I.D., Oke T.R. (2012). Local climate zones for urban temperature studies. Bull. Am. Meteorol. Soc..

[40] Final E.E.I. Plan Integral Para la Mejora de la Movilidad Y Plan del Centro de Ciudad de Panamá; Ciudad de Panamá, Alcaldía de Panamá y Banco Interamericano de Desarrollo (BID) 2017. https://dpu.mupa.gob.pa/wp-content/uploads/2017/06/20175-E.3-002-R01_INFORME_FINAL_ESTRATEGIAS_DE_MOVILIDAD_CH_PANAMA.pdf.

[41] Cold Feet, Warm Stripes|Londonist. https://londonist.com/2008/02/cold_feet_warm.

[42] Egri Á., Blahó M., Kriska G., Farkas R., Gyurkovszky M., Åkesson S., Horváth G. (2012). Polarotactic tabanids find striped patterns with brightness and/or polarization modulation least attractive: An advantage of zebra stripes. J. Exp. Biol..

[43] Jeong S.Y., Tso C.Y., Wong Y.M., Chao C.Y.H., Huang B. (2020). Daytime passive radiative cooling by ultra emissive bio-inspired polymeric surface. Sol. Energy Mater. Sol. Cells.

[44] AcryShield Ultra High Reflectance A590. https://www.nationalcoatings.com/acryshield-ultra-high-reflectance-a590.

[45] Tan Z., Deng X. (2017). Assessment of natural ventilation potential for residential buildings across different climate zones in Australia. Atmosphere.

[46] Energy2D—Interactive Heat Transfer Simulations for Everyone. https://energy.concord.org/energy2d/.

[47] Causone F. (2016). Climatic potential for natural ventilation. Archit. Sci. Rev..

[48] ISO—ISO 13786:2007—Thermal Performance of Building Components—Dynamic Thermal Characteristics—Calculation Methods. https://www.iso.org/obp/ui/#iso:std:iso:13786:ed-3:v2:en.

[49] Yi C.Y., Peng C. (2014). Microclimate Change Outdoor and Indoor Coupled Simulation for Passive Building Adaptation Design. Procedia Comput. Sci..

